# Clinical characteristics and risk factors of depression in prodromal Parkinson’s disease

**DOI:** 10.3389/fnins.2025.1708805

**Published:** 2026-01-12

**Authors:** Jianxia Xu, Yali Wang, Yuqian Li, Hui Wang, Weiguo Liu

**Affiliations:** 1 Department of Neurology, The Fourth Affiliated Hospital of Soochow University, Suzhou, China; 2 Department of Neurology, Affiliated BenQ Hospital of Nanjing Medical University, Nanjing, China; 3 Department of Neurology, Lianyungang Hospital of Traditional Chinese Medicine, Lianyungang Affiliated Hospital of Nanjing University of Chinese Medicine, Lianyungang, China; 4 Department of Neurology, The Affiliated Brain Hospital of Nanjing Medical University, Nanjing, China

**Keywords:** Parkinson’s disease, prodromal, depression, risk factors, anxiety

## Abstract

**Background:**

Depression is a highly prevalent non-motor symptom in Parkinson’s disease (PD) that can manifest several years prior to the clinical diagnosis of PD. The study was aimed to investigate the clinical characteristics and related risk factors of depression in prodromal PD (pPD) subjects.

**Methods:**

A total of 47 pPD participants from community population of East China and 39 healthy controls (HCs) were enrolled in the study. The pPD people were divided into two groups (pPD with depression and pPD without depression) according to the Diagnostic and Statistical Manual of Mental Disorders, Fifth Edition (DSM-V) criteria. The severity of depression was assessed via the 24-item Hamilton Depression Rating Scale (HAMD-24), and the clinical features of depression were assessed by calculating seven factors of the HAMD-24. Comparisons between the two pPD subgroups were conducted to evaluate the clinical characteristics of depression in the prodromal phase of PD. Risk factors for depression in prodromal PD were analyzed by multivariate Logistic regression analysis.

**Results:**

The prevalence of depression in prodromal PD was 25.53%. pPD group with depression (dpPD) had significantly less years of education and higher HAMD overall scores, Hamilton Anxiety Rating Scale (HAMA) scores, PD non-motor questionnaire scores compared with pPD without depression (ndpPD). The dpPD group obtained significantly higher scores than the ndpPD group across several domains, including anxiety/somatization, cognitive impairment, day/night changes, retardation, and despair. Multivariate Logistic regression analysis showed that anxiety was an independent risk factor for depression in pPD.

**Conclusion:**

Depression is common in prodromal PD and exhibit a multifaceted nature. pPD subjects with depression are vulnerable to comorbid with other non-motor symptoms. Anxiety is an established risk factor for ndPD, which indicates the critical value of early mood management in early disease intervention.

## Introduction

Parkinson’s disease (PD) is the second-most common progressive neurodegenerative disorder, which is characterized by motor manifestations and non-motor dysfunctions. Depression has been widely concerned due to its high prevalence and serious consequences. According to a recent meta-analysis research, the incidence of depression in all PD cases was up to 38%, which was about five times higher than the general population (Hemmerle et al., 2012; Cong et al., 2022). Depression can worsen motor dysfunctions, leading to severe disability and impaired living quality of patients (Ahmad et al., 2023). Thus, early identification and intervention of depressive symptoms in Parkinson’s disease are crucial for enhancing patients’ quality of life and slowing disease progression.

Notably, recent research has increasingly shifted focus to the prodromal stage of Parkinson’s disease (PD). The prodromal phase of PD (prodromal PD, pPD) refers to a stage where patients exhibit non-motor symptoms or even subtle motor symptoms, yet do not meet the clinical diagnostic criteria for PD (Postuma and Berg, 2019). Our previous community-based study found that the prevalence of probable pPD in our Chinese geriatric cohort was 2.1%, among whom 58.97% showed mild cognitive impairment (Pan et al., 2022). Accumulating evidence indicates that depressive symptoms may manifest up to two decades prior to PD diagnosis and be associated with an increased rate of PD (Gustafsson et al., 2015; Faustino et al., 2020). Indeed, depression is consistently included as a definitive prodromal marker for diagnosing the phase of prodromal PD (Berg et al., 2015; Heinzel et al., 2019). However, little is known about the features of depressive symptoms in prodromal PD.

The aim of the study was to examine the epidemiological profile, clinical characteristics, and risk factors of depressive symptoms in prodromal PD. We hope to provide evidence to guide early intervention strategies for PD patients with mood disorders.

## Methods

### Population

According to the Movement Disorder Society (MDS) research criteria for prodromal PD (pPD), the probability of a subject being in the pPD stage was calculated using the prior probability (the age-adjusted prevalence of pPD) and the combined likelihood ratios (LRs) of various disease risk markers and prodromal markers. A post-test probability score of ≥ 80% led to a diagnosis of probable pPD (Berg et al., 2015). We systematically incorporated feasible biomarkers suggested by the MDS research criteria and utilized a standardized structured questionnaire to screen for pPD people in the community. Risk markers were evaluated, including gender, occupational solvent/pesticide exposure, caffeine use, non-smoking history, family history, and abnormal hyperechogenicity of the substantia nigra (SN). Prodromal markers were assessed, including rapid eye movement sleep behavior disorder (RBD, polysomnographic or questionnaire-evaluated), mild motor symptoms, olfactory loss, constipation, excessive daytime somnolence, orthostatic hypotension, erectile dysfunction, urinary dysfunction, and depression/anxiety (Pan et al., 2022). All the pPD participants must meet the MDS research criteria for probable pPD. Patients were excluded if they (1) met the PD criteria suggested by the UK Parkinson’s Disease Society Brain Bank (Hughes et al., 1992); (2) were suspected to have parkinsonism-plus syndromes; (3) had dementia; (4) had major depressive disorder or other psychiatric diseases; (5) had other major systemic comorbidities. All participants were followed up for at least 5 years. We surveyed more than 3,000 elderly individuals in the community population of East China, and recommended subjects with suspected prodromal pPD be referred to the designated hospital for confirmation of diagnosis by two specialized neurologists. In addition, we recruited age-, sex-, and education-matched healthy controls (HCs) for comparison with pPD subjects. The study was approved by the Medical Ethics Committee of the Affiliated Brain Hospital of Nanjing Medical University.

### Clinical assessments

The diagnosis of pPD with depression (dpPD) was confirmed by two experienced psychiatrists according to the Diagnostic and Statistical Manual of Mental Disorders, Fifth Edition (DSM-V) criteria. To objectively measure depression severity, we employed the 24-item Hamilton Depression Rating Scale (HAMD-24) (Weintraub et al., 2006). In addition, the severity of motor symptoms was evaluated by the motor section of the Unified Parkinson’s Disease Rating Scale (UPDRS-III). Cognition was assessed using the Montreal Cognitive Assessment (MoCA). The Hamilton Anxiety of Scale (HAMA) was utilized to measure anxiety levels (He et al., 2024). Questionnaire-evaluated RBD was determined using the Rapid Eye Movement Sleep Behavior Disorder Questionnaire-Hong Kong (RBDQ-HK), with overall scale ≥ 17 or factor 2 of RBDQ-HK ≥ 8 indicating clinical RBD (Shen et al., 2014). Non-motor symptoms of PD were evaluated by the Parkinson’s disease Non-Motor Symptoms Questionnaire (PDNMS) (Ren et al., 2020; Chaudhuri et al., 2006).

### Statistical analysis

Statistical analyses were performed using SPSS Statistic 26.0. The normality of quantitative data was determined by using the Kolmogorov–Smirnov test. Normally distributed continuous variables were presented as mean ± standard deviation (SD), and the differences between two groups were compared using the two-sample *t*-test. Non-normally distributed data were presented as median (interquartile range), and the differences between two groups were analyzed with the Mann–Whitney *U* test. A chi-squared test was used to compare categorical variables such as gender and RBD. Multivariate logistic regression was conducted to identify risk factors associated with depression in pPD people. A two-tailed *p* < 0.05 was set as a threshold for statistical significance.

## Results

### Demographic and clinical characteristics

In this study, a total of 47 cases met the diagnostic criteria for prodromal PD and were enrolled in longitudinal follow-up. All participants completed standardized assessments at baseline and subsequent intervals. A total of 12 pPD participants were finally diagnosed with dpPD based on comprehensive evaluation. Thus, the prevalence of depressive disorders in pPD was 25.53%. Besides, 39 HCs were recruited in this study. There were no significant differences between pPD group and HCs group in terms of age, gender, and education. The pPD group had significantly higher scores in HAMD and HAMA, and lower scores in MoCA when compared to HCs group. RBD symptoms were significantly more frequent in pPD group than in HCs group (Table 1).

**Table 1:** Clinical data of prodromal Parkinson’s disease and healthy controls.

Characteristics	pPD (*n* = 47)	HCs (*n* = 39)	*p*-value
Age (years)	66(57, 69)	63(58.5, 65)	0.074^a^
Gender (male/female)	25/22	20/17	0.937^b^
Education (years)	10(8, 13)	10(9, 12)	0.931^a^
HAMD-24	8(3, 14)	1(0, 5)	0.000^a^^,*^
HAMA	5(3, 9)	2(0, 4)	0.000^a^^,*^
UPDRS-III	7.79 ± 4.69	–	–
PDNMS	9.36 ± 5.12	–	–
RBD (%)	34 (72.3%)	2(5.4%)	0.000^b^^,*^
MoCA	24(21, 25)	27(25, 28)	0.000^a^^,*^

pPD, prodromal Parkinson’s disease; HCs, healthy controls; HAMD-24, 24-item Hamilton Depression Rating Scale; HAMA, Hamilton Anxiety of Scale; UPDRS-III, the motor section of the Unified Parkinson’s Disease Rating Scale; PDNMS, Parkinson’s disease Non-Motor Symptoms Questionnaire; RBD, rapid eye movement sleep behavior disorder; MoCA, Montreal Cognitive Assessment.

a Mann-Whitney U test.

b Chi-squared test.

^*^p < 0.05.

### Comparisons between dpPD and ndpPD group

No significant differences were found in age, gender, UPDRS-III Cscores, frequency of RBD, and MoCA scores between dpPD and ndpPD group. The dpPD group had significantly less years of education and higher HAMA scores, PDNMS scores, and HAMD overall scores compared with ndpPD group. The 24-item HAMD can be divided into seven factor scores, including anxiety/somatization (items 10–13, 15, 17), weight loss (item 16), cognitive impairment (items 2, 3, 9, 19–21), day/night changes (item 18), retardation (items 1, 7, 8, 14), sleep disturbance (items 4–6), and despair (items 22–24) (Zhao et al., 2024). The dpPD group exhibited significantly higher scores in the factor of anxiety/somatization, cognitive impairment, day/night changes, retardation, and despair compared to the ndpPD group. There were no significant differences between the two groups in weight loss and sleep disturbance (see Table 2).

**Table 2:** Clinical data of prodromal Parkinson’s disease with or without depression.

Characteristics	dpPD (*n* = 12)	ndpPD (*n* = 35)	*p*-value
Age (years)	65 (56.75, 68.00)	67.00 (57.00, 70.00)	0.293^a^
Gender (male/female)	6/6	19/16	0.797^b^
Education (years)	7.50 (6.25, 11.75)	11.00 (9.00, 14.00)	0.049^a^^,*^
HAMA	10.00 (8.00, 12.00)	4.00 (2.00, 7.00)	0.001^a^^,*^
UPDRS-III	9.75 ± 4.16	7.11 ± 4.73	0.093^c^
PD-NMS	12.83 ± 5.02	8.17 ± 4.64	0.005^c^^,*^
RBD (%)	7 (58.3%)	22 (62.9%)	0.781^b^
MoCA	22.00 (18.00, 24.75)	24.00 (21.00, 25.00)	0.157^a^
HAMD-24	16.00 (14.25, 20.75)	5.00 (2.00, 10.00)	0.000^a^^,*^
Anxiety/Somatization	5.50 (3.25, 7.50)	1.00 (0.00, 3.00)	0.000^a^^,*^
Weight loss	0.00 (0.00, 0.00)	0.00 (0.00, 0.00)	0.403^a^
Cognitive impairment	4.00 (2.25, 4.75)	1.00 (0.00, 1.00)	0.000^a^^,*^
Day/night changes	0.00 (0.00, 1.00)	0.00 (0.00, 0.00)	0.003^a^^,*^
Retardation	4.00 (3.00, 4.00)	1.00 (0.00, 2.00)	0.000^a^^,*^
Sleep disturbance	3.00 (1.00, 4.00)	2.00 (0.00, 3.00)	0.133^a^
Despair	2.00 (1.00, 2.75)	1.00 (0.00, 1.00)	0.000^a^^,*^

dpPD, prodromal Parkinson’s disease with depression; ndpPD, prodromal Parkinson’s disease without depression; HAMA, Hamilton Anxiety of Scale; UPDRS-III, the motor section of the Unified Parkinson’s Disease Rating Scale; PDNMS, Parkinson’s disease Non-Motor Symptoms Questionnaire; RBD, rapid eye movement sleep behavior disorder; MoCA, Montreal Cognitive Assessment; HAMD-24, 24-item Hamilton Depression Rating Scale.

a Mann-Whitney U test.

b Chi-squared test.

c Two-sample *t*-test.

^*^p < 0.05.

### Result analysis by multivariate logistic regression

Taking candidate factors with statistically significant differences between dpPD and ndpPD group as independent variables, we performed binary logistic regression analysis. The HAMA scores which assessed anxiety severity were confirmed to be an independent risk factor for the occurrence of depression in prodromal PD people (OR = 0.755, 95%CI: 0.574–0.993, *p* = 0.044) (see Table 3).

**Table 3:** Logistic regression analysis of risk factors for depression in prodromal PD.

Variables	*p*-value	OR	95%CI
Education	0.252	1.110	0.929–1.326
HAMA	0.044*	0.755	0.574-0.993
PDNMS	0.926	0.989	0.781–1.252

HAMA, Hamilton Anxiety of Scale; PDNMS, Parkinson’s disease Non-Motor Symptoms Questionnaire; OR, Odds Ratios; 95%CI, 95% Confidence Intervals.

^*^p < 0.05.

## Discussion

The present study investigated the characteristics of depressive symptoms in prodromal PD people and explored the potential risk factors for the incidence of depression in the early stage. We found that depressed prodromal PD subjects had fewer years of education, more non-motor symptoms, and were vulnerable to anxiety. More importantly, anxiety may facilitate the presence of depression in the prodromal phase of PD.

Depression is a common comorbid symptom for PD, and a previous cohort study found that patients with prior diagnosis of depression exhibited a higher risk of developing PD and the association remained significant over more than 2 decades (Gustafsson et al., 2015). Consequently, depressive symptoms are now recognized as one of the key prodromal markers of PD (Heinzel et al., 2019). Previous studies showed that depression preceded motor symptoms in approximately 30% of PD cases and was the initial symptom in 12–22% of patients (Postuma et al., 2012). We found that 25.53% of population with prodromal PD exhibited depression in our cohort. Several hypotheses may possibly reveal the underlying mechanisms. According to the “Braak hypothesis”, at Braak stage II, serotonergic dysfunction in the raphe nuclei precedes dopaminergic degeneration which happens at Braak stage III, which may contribute to the emergence of depressive symptoms in prodromal PD (Braak et al., 2003; Wang et al., 2018). The serotonin hypothesis proposes that serotonergic activity reduced in early PD to compensate for dopaminergic deficits through its inhibitory effect on striatal dopamine release. This compensatory mechanism may explain why depressive symptoms precede motor manifestations (Schuurman et al., 2002).

Prior studies indicated that depression served as a marker for a severe overall non-motor burden in early PD (Swallow et al., 2016; Pont-Sunyer et al., 2015). Patients with late-onset depression were shown to exhibit dopaminergic deficit in the striatum resembling those observed in PD and present more motor and non-motor dysfunction seen in PD, including rigidity, bradykinesia, apathy, sleep disorders, cognitive impairment, and RBD (Kazmi et al., 2021). Similarly, our study found that prodromal PD subjects frequently presented with comorbid depression, RBD, more severe anxiety symptoms and poorer cognitive function. Moreover, depressed prodromal PD subjects showed significantly more severe anxiety symptoms and overall non-motor manifestations compared to non-depressed prodromal PD cases.

Prodromal PD with depression was less educated than those without depression in our study, consistent with previous evidence (Cong et al., 2022; Hakulinen et al., 2019; Baiano et al., 2020). As established in prior studies, educational attainment which reflects cognitive reserve enables individuals to confer resistance to neurodegenerative processes and exerts a protective effect against depression occurrence by modulating locus coeruleus noradrenergic activity (Stern, 2012; Gao et al., 2025; Robertson, 2013).

To our knowledge, our study was the first report demonstrating the clinical features of depression in prodromal PD via the 7 sub-factors of HAMD-24 items in detail. Anxiety/ somatization was the most prominent manifestation, aligning with the prior study on definitively diagnosed PD patients (Lian et al., 2018). Depressive symptoms were also characterized by a higher burden of cognitive disturbance, circadian fluctuations, retardation, and despair. Depression was confirmed to be associated with longitudinal cognitive decline in early PD patients, potentially mediated by the disruption of the prefrontal-limbic axis (Ng et al., 2015; Surdhar et al., 2012). In addition, the Multivariate logistic regression showed that anxiety was the independent influencing factor of depression in prodromal PD. Notably, depression and anxiety frequently coexist in Parkinson’s disease patients, with comorbidity rates as high as 14–50% (Landau et al., 2016; Dissanayaka et al., 2010), attributing to the same neurobiological substrate of serotonergic degeneration (Nuti et al., 2004). This finding supports the implementation of anxiety-focused interventions as a potential strategy to delay depressive onset in high-risk prodromal PD populations.

The current study has several limitations. First, the MDS diagnostic research criteria for prodromal PD in MDS are probability-based, and the sensitivity and specificity vary considerably across different validation studies (Fereshtehnejad et al., 2017; Mahlknecht et al., 2016; Pilotto et al., 2017). The results may change with future revisions of the criteria. Second, cross-sectional design precludes causal inferences about anxiety-depression relationships. Third, the relatively small sample size in this study might limit the generalizability of our findings, and future studies with larger cohorts and longitudinal follow-up are necessary to validate these results.

## Conclusion

In summary, our study explored the characteristics of depression in the prodromal phase of Parkinson’s disease and suggested that depression is a common symptom, frequently coexisting with other non-motor symptoms and manifesting in multiple domains. Anxiety is an independent risk factor for the development of depression in prodromal PD, and early intervention for mood symptoms may potentially help prevent the progression of the disease.

## Data Availability

The original contributions presented in the study are included in the article/supplementary material, further inquiries can be directed to the corresponding author.
